# Tracheal Stenosis Identified With Bronchoscopy Nine Years After Emergent Short-Term Intubation

**DOI:** 10.7759/cureus.110330

**Published:** 2026-06-05

**Authors:** Sanjana Apte, Harshavardhan Senapathi, Michael Hsu, Boyd Hehn

**Affiliations:** 1 Anesthesiology, Guthrie Robert Packer Hospital, Sayre, USA; 2 General Surgery, Guthrie Robert Packer Hospital, Sayre, USA; 3 Pulmonary and Critical Care Medicine, Guthrie Robert Packer Hospital, Sayre, USA

**Keywords:** a-frame deformity, endotracheal intubation, fiberoptic bronchoscopy, tracheal balloon dilation, tracheal stenosis

## Abstract

Tracheal stenosis is an uncommon but life-threatening complication of endotracheal intubation. The prevalence of tracheal stenosis after long-term intubation is 19%; however, only 1-2% of patients are symptomatic. Common risk factors include a history of previous intubation, duration of intubation, difficult airway, traumatic intubation, prior tracheostomy, advanced age, female sex, estrogen use, severe respiratory failure, and severe gastroesophageal reflux disease, among others. The typical presentation of tracheal stenosis is gradual dyspnea and wheezing that is unresponsive to bronchodilators and steroids. We describe a case of a 33-year-old female patient with atypical presentation who was diagnosed with tracheal stenosis on bronchoscopy nine years following initial short-term endotracheal intubation. Clinicians should maintain a high index of suspicion for tracheal stenosis and a low threshold to perform fiberoptic bronchoscopy in patients with persistent respiratory symptoms after a history of endotracheal intubation, to avoid delays in diagnosis and treatment.

## Introduction

Tracheal stenosis is an uncommon but life-threatening complication of endotracheal intubation first described in the literature in 1880. The prevalence of tracheal stenosis after long-term intubation is 19%; however, only 1-2% of patients are symptomatic. The most common location of tracheal stenosis is at the site of the endotracheal tube (ETT) cuff; however, it can occur anywhere from the tip of the ETT to the level of the glottis. The area of the trachea that is affected is usually 2 cm long and involves the anterior and lateral walls. The posterior wall is relatively protected from stenosis because of its ability to distend into the esophagus [1]. The etiology of stenosis is thought to be localized ischemia from the pressure of the ETT cuff. The risk is higher if the ETT cuff pressures are more than 20 mmHg. Smaller ETT sizes can also lead to higher cuff pressure requirements to prevent an air leak [2]. Therefore, the thought is that patients with prolonged intubations are at higher risk for tracheal stenosis given the increased duration of cuff pressure on the tracheal mucosa. Other proposed mechanisms of this injury include mucosal destruction caused by erosion of the ETT tip and excessive patient movement after intubation.

Other risk factors for the development of tracheal stenosis include traumatic intubation, history of previous intubation, duration of intubation, history of previous tracheostomy, advanced age, female sex, estrogen use, severe respiratory failure, severe gastroesophageal reflux disease, Wegener's granulomatosis, sarcoidosis, obstructive sleep apnea, and previous radiation exposure to the airway [2]. Pregnancy is also associated with increased mucosal edema and friability in the upper airway that can manifest as early as the first trimester and predispose to tracheal injury [3].

Endotracheal intubation can also lead to various other airway complications, including laryngeal edema, mucosal ulceration, and granulation formation. The typical presentation of tracheal stenosis is gradual dyspnea and wheezing that is unresponsive to bronchodilators and steroids. Respiratory failure is rarely the presenting symptom.

## Case presentation

A 33-year-old female with past medical history of cigarette smoking (0.5 packs per day since age 13), mood disorder, and insomnia was brought in by emergency medical services (EMS) for a suicidal attempt with medication overdose (amitriptyline and clonazepam). Per EMS, the patient was unresponsive to a sternal rub. The initial attempt at endotracheal intubation by EMS was unsuccessful. The patient was successfully intubated in the emergency room with a 7.5-mm-sized ETT and noted to have vocal cord swelling at the time of intubation, though there was no prior history of hoarseness or change of voice. She was subsequently admitted to the intensive care unit, where she was extubated after 24 hours. One year later, she presented to her primary care provider's office for evaluation of dyspnea and chronic cough, ongoing since the time of extubation. For the next nine years, she continued to have multiple subsequent visits with medical providers for similar respiratory symptoms and difficulty swallowing. During this period, she was treated with short and long-acting beta agonists as well as corticosteroids with intermittent resolution of her symptoms.

Her first computed tomography (CT) scan of the chest and pulmonology evaluation was at the beginning of the COVID-19 pandemic, four years after her initial intubation. A chest CT at that time was reported as unremarkable on initial radiology review; however, moderate to severe tracheal stenosis was noted during independent review by a pulmonologist one to two years after the CT scan was performed (Figure 1). 

**Figure 1:**
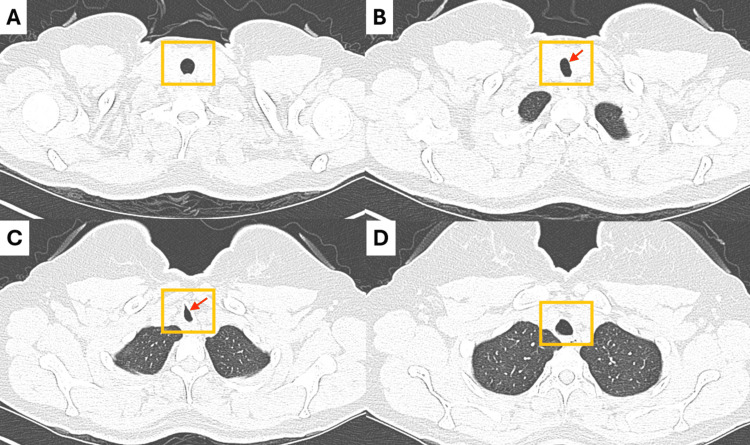
CT scan of the chest (lung windows, axial slices) four years following intubation, demonstrating the trachea (yellow box) with areas of tracheal stenosis (red arrow). Panels A to D represent cranial to caudal.

Amid the COVID-19 pandemic, the patient was unfortunately unable to complete pulmonary function testing (PFT) with methacholine challenge. She was given a diagnosis of asthma- chronic obstructive pulmonary disease (COPD) overlap syndrome, given her active cigarette smoking and good clinical response to bronchodilators and corticosteroids. It was not until the subsequent review of her CT scan by an independent pulmonologist that the concern for tracheal stenosis was documented, as a contributory factor to her respiratory symptoms in concurrence with COPD. PFTs demonstrated moderate obstruction consistent with Global Initiative for Chronic Obstructive Lung Disease (GOLD) stage II COPD [4]. Chest CTs from five years (Figure 2), seven years (Figure 3), eight years (Figure 4), and nine years (Figure 5) following her initial intubation were again reviewed, and she was noted to have moderate to severe tracheal stenosis on both imaging studies, likely originating from the initial intubation. 

**Figure 2:**
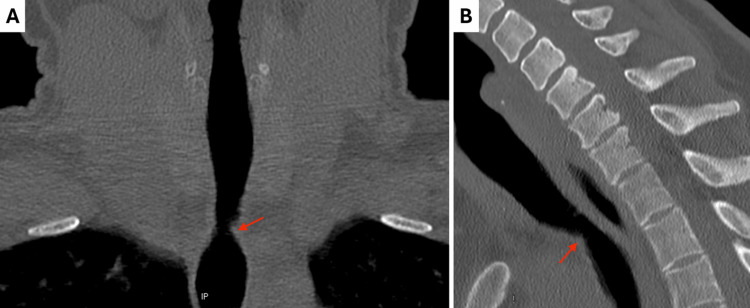
CT scan of the cervical spine, bone windows, coronal (A) and sagittal (B) slices, five years following intubation, demonstrating tracheal stenosis (red arrow).

**Figure 3:**
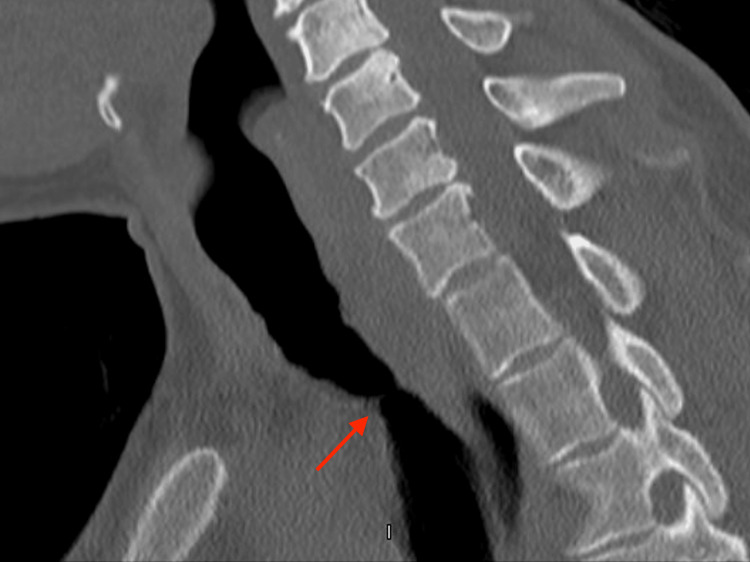
CT scan of the cervical spine, bone window, sagittal slice, seven years following intubation, demonstrating tracheal stenosis (red arrow).

**Figure 4:**
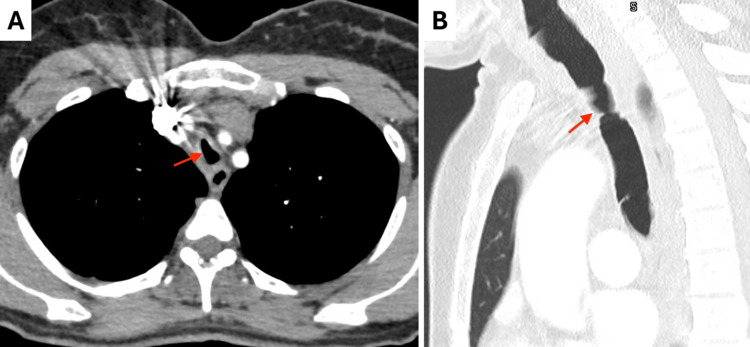
CT scan of the chest (A: soft tissue window, axial slice; B: lung window, sagittal slice), eight years following intubation, demonstrating tracheal stenosis (red arrow).

**Figure 5:**
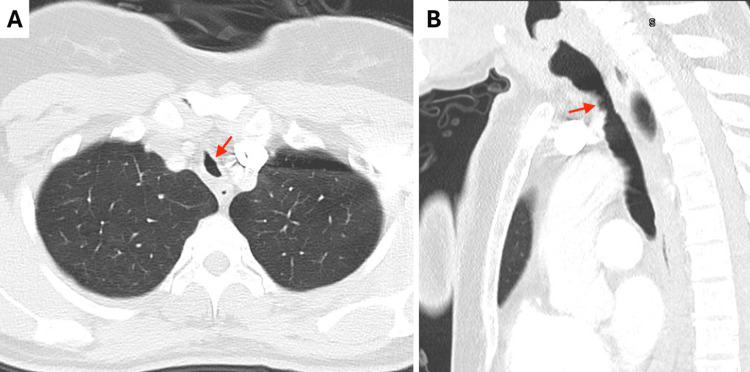
CT scan of the chest, lung window (A: axial slice; B: sagittal slice), nine years following intubation, demonstrating tracheal stenosis (red arrow).

Despite having suspected tracheal stenosis on her CT imaging, the patient underwent two separate surgical procedures, again requiring endotracheal intubation. No ventilation or oxygenation issues were noted during either surgery. Ultimately, at nine years following her initial intubation, she underwent bronchoscopy, which demonstrated hyperemic beefy arytenoids and ratty posterior commissure suggestive of laryngopharyngeal reflux. In the cervical trachea, starting two rings below the cricoid cartilage, there was an A-frame deformity that became moderately severe two rings further below, associated with a cicatricial scar (Figure 6).

**Figure 6:**
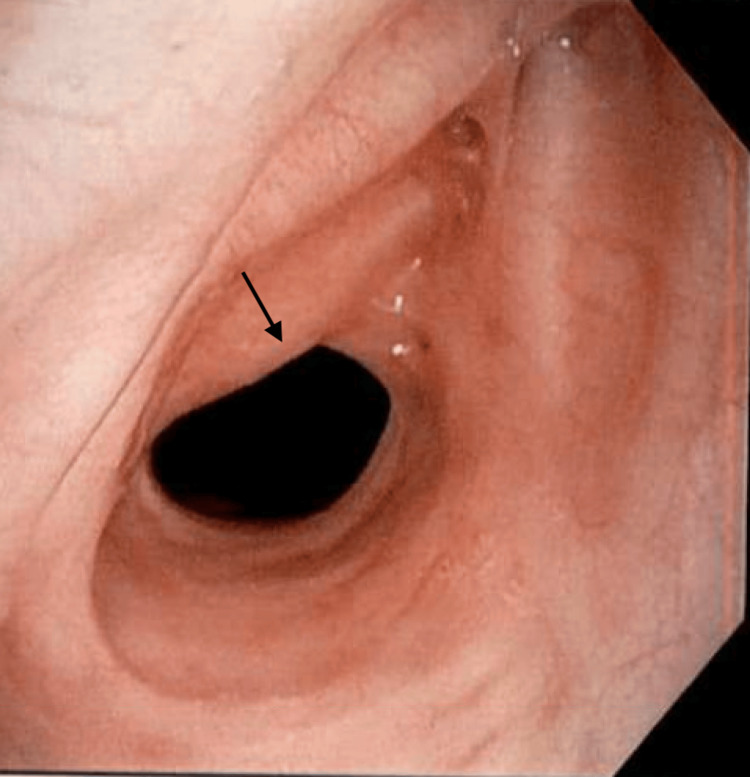
Bronchoscopic findings demonstrating tracheal stenosis with A-frame deformity (black arrow).

With a cough, there was occlusion of the trachea at the A-frame deformity. The region of maximal stenosis was one ring in vertical height. When the bronchoscope passed through the stenosis, the patient developed stridor, but was able to tolerate the bronchoscopy. The estimated diameter was 7-8 mm at the region of maximal stenosis. She was scheduled for another bronchoscopy with general anesthesia for laser or dilation of the tracheal stenosis. She was also started on a proton-pump inhibitor and scheduled for a barium swallow given the laryngopharyngeal reflux. 

The following month, she underwent bronchoscopy with holmium laser destruction and tracheal balloon under general anesthesia. The airway examination revealed tracheal stenosis three rings distal to the cricoid cartilage, an A-frame deformity, and an anterior cicatrix at the fourth ring. The tracheal diameter was estimated to be less than 8 mm. The bronchoscope crossed the area of stenosis, and the bilateral airway examination was normal through the segmental airways. Holmium laser was initially used at 20 W and then decreased to 15 W for the destruction of cicatrix. After several pulses with the proximal cicatrix released, it was clear there was a distal fixed stenosis that did not release, and no further lasering was done to prevent cartilage injury. Balloon dilation was then performed sequentially at 8 mm, 9 mm, and then 10 mm, all for 60 seconds at each measure, dropping the balloon between dilations to allow for ventilation. Subsequently, balloon dilation was performed sequentially at 13 mm and then 14 mm, for 60 seconds at each measure, dropping the balloon between dilations to allow for ventilation. Final dilation to 15 mm was performed for 120 seconds. Post-dilation, there was improvement in the tracheal lumen diameter at the region of maximal stenosis, but less than expected due to the fixed distal stenosis. Ultimately, the patient's symptoms completely resolved after laser destruction and tracheal balloon dilation. 

## Discussion

Tracheal stenosis is a rare airway complication following short-term endotracheal intubation. The patient described in this report was only intubated for 24 hours. The risk factors for tracheal stenosis in our patient were female gender and likely trauma during the initial unsuccessful intubation attempt by EMS. The patient had a significant cigarette smoking history that delayed the diagnosis of tracheal stenosis. An appropriate diagnosis of tracheal stenosis, especially in patients with lower degrees of stenosis, is sometimes difficult, and the literature indicates that 44% of patients are often incorrectly treated for asthma or chronic bronchitis [1]. 

Exertional dyspnea is the most common presenting symptom of tracheal stenosis and is usually evident when the tracheal lumen becomes less than 8 mm. Symptoms may appear at rest when the tracheal lumen is narrowed to 5 mm or less. Our patient first noted dyspnea on exertion four years after her initial intubation. Ultimately, during bronchoscopy, the tracheal lumen was estimated to be 7-8 mm in diameter. Nesek-Adam et al. reported 12% incidence of laryngeal stenosis in patients with tracheal intubation for 11 days or longer, 5% incidence for intubation between six and 10 days, and a 2% incidence for intubation for less than six days [1]. Songu and Ozkul concluded that patients who were previously intubated for more than 48 hours were at higher risk of developing tracheal stenosis [5]. Our patient’s intubation period was less than 24 hours, resulting in a moderately severe A-frame deformity, an exceedingly rare occurrence given the short intubation duration. In this case, tracheal stenosis was more likely to have occurred from traumatic intubation and not the longevity of intubation.

Rubio et al. reported on a 28-year-old female patient who had an ETT for 72 hours, which resulted in circumferential stenosis decreasing the tracheal lumen to 4 mm [6]. Xue et al. reported on a 64-year-old female patient who remained intubated for 24 hours following an oral surgery procedure [7]. She developed stridor and breath-holding one day after extubation; a chest CT on postoperative day 4 revealed cervical tracheal stenosis, and symptoms improved after tracheotomy. Li et al. reported on a 65-year-old male who was found to have tracheal stenosis after 28 hours of intubation for an acute exacerbation of COPD [8]. 

Our patient also had a delayed diagnosis and treatment of her tracheal stenosis symptoms, given her comorbidity of asthma-COPD overlap syndrome and ongoing cigarette smoking. In fixed upper-airway obstructions, airflow is generally limited equally during both inspiration and expiration due to the lesion maintaining a constant caliber. As such, the classic flow-volume loop finding is a "box pattern" due to plateauing of both the inspiratory and expiratory limbs. Our patient's symptoms were also intermittently responsive to inhaled bronchodilators and oral corticosteroids, which further lowered suspicion for tracheal stenosis. Zubairi et al. reported a similar case of tracheal stenosis mimicking asthma in a 62-year-old female who presented to the emergency room with severe dyspnea and was diagnosed with status asthmaticus [2]. However, due to the severity and persistence of her symptoms impeding her discharge, she underwent bronchoscopy as an inpatient and was found to have tracheal stenosis in an expeditated timeline, contrary to our patient.

## Conclusions

Tracheal stenosis is an uncommon but life-threatening complication of endotracheal intubation and can occur anywhere from the tip of the ETT to the level of the glottis. The typical presentation is gradual dyspnea and wheezing that is unresponsive to bronchodilators and steroids, with respiratory failure rarely being the presenting symptom. Our case exemplifies that clinicians should maintain a high index of suspicion for tracheal stenosis along with a low threshold to perform fiberoptic bronchoscopy in patients with persistent respiratory symptoms after a history of endotracheal intubation, despite minimal risk factors for tracheal stenosis, to prevent delays in diagnosis and treatment. Furthermore, we highlight the implications of missed opportunities to diagnose and treat this complication earlier. Modalities to manage tracheal stenosis in such patients may include holmium laser destruction and tracheal ballooning.
